# Supplementary material for: Risk factors for malaria-related mortality among children under five at Mbale Regional Referral Hospital, Uganda, 2020–2024: A case-control study
Source: PLOS Glob Public Health. 2026 Apr 20;6(4):e0006308. doi: 10.1371/journal.pgph.0006308 (PMC13094984; doi:10.1371/journal.pgph.0006308)
Supplement: Attachment — Submitted filename: Mbale Regional Hospital Study Review.docx [file pgph.0006308.s002.docx]

**Review Summary Statement**

Manuscript Number: PGPH-D-25-03409

Title: Risk factors for malaria-related mortality among children under five at Mbale Regional Referral Hospital, Uganda, 2020-2024: a case-control study

General Assessment:

This manuscript addresses a critical public health priority: reducing malaria-related mortality among children under five in high-burden settings. The authors present a retrospective case-control study conducted at Mbale Regional Referral Hospital, a region with high endemicity. The study successfully identifies clinically relevant predictors of mortality, specifically neurological complications (convulsions, loss of consciousness), severe anaemia, and delays in care-seeking. The findings are actionable and may reinforce the necessity of early recognition of danger signs and prompt strengthening of referral pathways. The use of a 1:2 unmatched case-control design with systematic sampling is methodologically appropriate for this research question.

However, despite these strengths, there are significant methodological and compliance concerns that must be addressed before the manuscript is suitable for publication. These primarily concern the precision of the estimates, the stability of the multivariable model, and adherence to the journal’s data availability policy, as well as the action plan taken after identification of the risk factors, this being sanctioned at policy level.

**Major Points for Revision:**

1. Statistical Precision and Confidence Intervals:

The adjusted odds ratios (aORs) for key risk factors exhibit extremely wide 95% confidence intervals, particularly for neurological complications. For instance, loss of consciousness shows an aOR of 14.0 with a CI of 1.41–113, and convulsions show a CI of 4.2–71. These wide intervals suggest a lack of statistical power or precision, limiting the reliability of the point estimates. The authors should discuss this limitation explicitly and consider if the sample size was sufficient for these specific stratifications.

2. Multivariable Model Selection and Variable Exclusion:

There is a notable discrepancy between the bivariate and multivariable analyses regarding cerebral malaria and overall danger signs. Both were highly significant in the bivariate analysis (p<0.0001) but do not appear to be retained or significant in the final model. Given that "convulsions" and "loss of consciousness" (components of cerebral malaria) remained significant, there is a concern regarding potential multicollinearity that may have obscured the impact of these broader categories. A more transparent explanation of the model building and variable selection process is required.

3. Data Availability Policy Compliance:

The current Data Availability Statement ("The datasets... are not publicly available" ) does not appear to comply with PLOS Global Public Health’s requirements for open data. Unless legal or ethical restrictions are explicitly detailed and approved by the editors, the data underlying the findings must be made available without restriction. The authors must revise this statement or provide a compelling justification for an exemption.

4. Retrospective Data Limitations:

While the authors acknowledge the use of secondary data, the implications of this on the "time to care" variables should be further critically evaluated. Retrospective records are often prone to significant recall or recording bias regarding the exact timing of symptom onset, which is a central finding of this study (>24 hours delay aOR = 8.8).

5. Usage of Study Findings

The authors alluded to the fact that this was a public health emergency as a justification to conduct this research. It is therefore imperative to give insights into how the results of this study has helped shape the response plans, at least at the same facility

Decision: Major Revision

Based on the clinical relevance of the findings but the substantial need for statistical clarification and policy compliance, I recommend a Major Revision.
