# Supplementary material for: Risk factors for malaria-related mortality among children under five at Mbale Regional Referral Hospital, Uganda, 2020–2024: A case-control study
Source: PLOS Glob Public Health. 2026 Apr 20;6(4):e0006308. doi: 10.1371/journal.pgph.0006308 (PMC13094984; doi:10.1371/journal.pgph.0006308)
Supplement: Attachment — Submitted filename: Response to reviewers commments-Kwizera.docx [file pgph.0006308.s004.docx]

January 5, 2026

Dr. Collins Otieno Asweto, PhD

Academic Editor

PLOS Global Public Health

**RE: Risk factors for malaria-related mortality among children under five at Mbale Regional Referral Hospital, Uganda, 2020–2024: a case-control study**

Dear, Dr. Collins Otieno Asweto, PhD

We sincerely thank the reviewers and the Editor for their thorough review and constructive remarks. We have carefully considered and addressed all comments in the revised manuscript.

A detailed point-by-point response to the reviewers’ comments and Editor are attached, with corresponding revisions highlighted in the tracked-changes and clean versions of the manuscript.

We acknowledge the efforts of the reviewers and the Editor. For any further clarifications and information, please don’t hesitate to contact us.

Sincerely,

Patrick Kwizera (Corresponding Author)

**RESPONSE TO REVIEWERS’ COMMENTS**

**Reviewer #1 comments**

**Comment # 1**

The author(s) states that they used secondary data that required no ethical review board, yet chooses not to attach it, at least for review process. I have attached my comments in the document for further insights

**Response**

Thank you for this observation, I have shared the data set in the manuscript

**Comment # 2**

Statistical Precision and Confidence Intervals:

The adjusted odds ratios (aORs) for key risk factors exhibit extremely wide 95% confidence intervals, particularly for neurological complications. For instance, loss of consciousness shows an aOR of 14.0 with a CI of 1.41–113, and convulsions show a CI of 4.2–71. These wide intervals suggest a lack of statistical power or precision, limiting the reliability of the point estimates. The authors should discuss this limitation explicitly and consider if the sample size was sufficient for these specific stratifications.

**Response**

Thank you for this observation. We acknowledge that some adjusted odds ratios, particularly for neurological complications such as convulsions and loss of consciousness, have wide 95% confidence intervals. This reflects limited **statistical precision,** likely due to the relatively small number of events in certain subgroups rather than errors in analysis.

We have explicitly discussed this limitation in the revised manuscript, noting that the estimates for these variables should be interpreted with caution. While the overall sample size (300 participants) was sufficient to detect associations for the primary outcomes, certain stratified analyses had fewer cases, which contributed to the wide confidence intervals. This limitation is now highlighted in the **Limitation section, see lines (266-269)**

**Comment #3**

**Multivariable Model Selection and Variable Exclusion:**

There is a notable discrepancy between the bivariate and multivariable analyses regarding cerebral malaria and overall danger signs. Both were highly significant in the bivariate analysis (p<0.0001) but do not appear to be retained or significant in the final model. Given that "convulsions" and "loss of consciousness" (components of cerebral malaria) remained significant, there is a concern regarding potential multicollinearity that may have obscured the impact of these broader categories. A more transparent explanation of the model building and variable selection process is required.

**Response**

Thank you for the observation, we have clarified the model-building process in Methods:
“Variables significant at p<0.2 in bivariate analysis were considered for multivariable logistic regression. We assessed multicollinearity using the Variance Inflation Factor (VIF), with all variables showing VIF <5, indicating no significant multicollinearity. Variables such as cerebral malaria was excluded from the final model to avoid collinearity with component neurological features, specifically convulsions and loss of consciousness, which were retained as independent predictors see lines (157-159)

**Comment #4**

**Data Availability Policy Compliance:**

The current Data Availability Statement ("The datasets... are not publicly available" ) does not appear to comply with PLOS Global Public Health’s requirements for open data. Unless legal or ethical restrictions are explicitly detailed and approved by the editors, the data underlying the findings must be made available without restriction. The authors must revise this statement or provide a compelling justification for an exemption.

**Response**

We thank you for your observation. We have made dataset has been made available in the manuscript

**Comment #5**

**Retrospective Data Limitations:**

While the authors acknowledge the use of secondary data, the implications of this on the "time to care" variables should be further critically evaluated. Retrospective records are often prone to significant recall or recording bias regarding the exact timing of symptom onset, which is a central finding of this study (>24 hours delay aOR = 8.8).

**Response**

We agree with this concern. Timing variables derived from retrospective records are susceptible to documentation and recall bias. We have strengthened the Limitations section to explicitly discuss potential misclassification of symptom onset times and how this could affect the magnitude of associations observed for delayed care-seeking, while noting that the strong association observed is consistent with existing literature, see lines (260-264)

**Comment #6**

**Usage of Study Findings**

The authors alluded to the fact that this was a public health emergency as a justification to conduct this research. It is therefore imperative to give insights into how the results of this study has helped shape the response plans, at least at the same facility

**Response**

We appreciate this recommendation. We have added a paragraph in the Discussion describing how findings will informed facility-level response actions, including reinforcement of triage for neurological danger signs, prioritization of rapid referral and admission, and target staff training, illustrating how study findings can inform interventions to reduce pediatric malaria mortality, see lines (282-285)

**Reviewer # 2 comments**

**Comment #1**

The study uses an unmatched 1:2 case–control design appropriate for evaluating mortality-related factors in severe malaria, with case and control definitions aligned with WHO criteria for reliability and comparability. Controls were systematically sampled from a large frame of over 32,000 survivors, reducing bias. Sample size was justified using an accepted approach, with 100 cases clearly explained. Data collection was robust, employing standardized tools, trained abstractors, and daily checks. Inclusion and exclusion criteria were explicit, and operational definitions for clinical variables were consistent. The multivariable logistic regression model was appropriate, including predictors significant at the bivariate level, with adjusted odds ratios and 95% CIs reported.
The associations like neurological signs, severe anemia, vomiting, and delayed care align with known predictors of severe malaria and death in low-resource settings. These findings support the importance of early recognition and timely care, consistent with cited literature.
Statistical analyses are appropriate. Descriptive statistics clearly characterize the groups. Chi-square tests are suited for comparing categorical variables. Logistic regression is correctly employed to control confounders.

**Response**

We thank you for this positive feedback

**Comment #2**
Several variables in Table 1 display inconsistencies or reversal of percentages (e.g., severe anaemia appears very common in both cases and controls, but proportions differ between the text and the table). A thorough review of the table with clear labels is necessary.

**Response**

Thank you for your observation, the table has been thoroughly reviewed.

**Comment #3**

Some confidence intervals are very wide (e.g., aOR for loss of consciousness), reflecting limited precision and possibly sparse data, and this should be acknowledged.

**Response**

Thank you for this observation. We have revised the manuscript to acknowledge this in the Limitations section, (Line 266-269)

**Comment #4**

The manuscript should explicitly mention checks for multicollinearity and model fit

**Response**

We have revised the Methods section to explicitly state that multicollinearity was assessed using variance inflation factors (VIFs), and model fit was evaluated using standard logistic regression diagnostics. No problematic collinearity was observed among retained variables (Lines 157-158)

**Comment #5**

Continuous variables were dichotomized; a rationale should be provided because categorization may reduce statistical power.

**Response**

We thank you for this observation. Continuous variables, such as age, were categorized using clinically meaningful cut-offs to facilitate interpretation and comparison across subgroups (though categorization may reduce statistical power)

**Comment #6**

Because delays in care and clinical severity are interrelated, discussion of possible confounding or mediation would strengthen the analysis.

**Response**

Thank you for this guidance: We acknowledge that delays in care and clinical severity are interrelated, and some of the observed association between delayed presentation and mortality may reflect more severe disease at admission. This clarification has been added to the Discussion section Lines

**Comment #7**

The manuscript indicates that data were regularly collected, de-identified, and used with institutional approval. To comply with PLOS Global Public Health’s mandatory data availability policy, the authors are required to provide: A Data Availability Statement indicating where the dataset can be accessed publicly (repository link), or A formal explanation is provided when data cannot be shared, such as due to legal or ethical restrictions.

**Response**

We have ensured that the relevant data (data set) are now included and accessible in the manuscript.

**Comment #8**

The manuscript claims that the work was “not human subjects research” while also stating that “all experimental protocols were approved by the US CDC human subjects review board,” which is contradictory. The authors should clarify whether IRB review occurred or was waived due to the non-research classification.

**Response.**

Thank you for your observation, this was non-research and did not require IRB approval and it has been clarified in the manuscript under ethical consideration.

**Comment #9**

No concerns about dual publication or research misconduct are evident.
The manuscript is generally well written, clear, and organized. It uses standard, understandable English. The sections follow a logical sequence, variables are defined, and interpretations are concise.

**Response.**

Thank you for the positive feedback

**Comment #10**

Some typographical errors (spacing, punctuation) should be corrected.

**Response.**

Thank you for your observations these have been addressed throughout the manuscript

**Comment #11**

Proper table formatting is essential for aligning rows and values effectively.

**Response.**

Thank you, the proper table alignment has been done through out the manuscript

**Comment #12**

A few sentences in the Discussion reiterate points already made, tightening these would enhance readability.

**Response**

Thank you for your observation, The Discussion has been edited to remove redundant statements and improve clarity and conciseness.

**Reviewer #3 comments**

**Comment #1**

The manuscript is insightful and very informative. However, the authors should please note the following and response as appropriate:
Kindly check the punctuation for line 178 to 180 and rectify accordingly. It is ambiguous.

**Response**

Thank you for your observation, this has been made clearer in the manuscript see Lines (178-180)

**Comment #2**

Please, check and rewrite the results section for the socio-demographics and clinical characteristics (from line 178 to 191, especially), and make sure the format and figures are consistent and accurate. For example, the table presents 62(62%) cases and 22(11%) control with convulsion, but the table description says 42% vs. 10%. Very confusing, please where you got the 42% vs. 10% from. Similarly, with cerebral malaria, the table says 42(42%) cases and 3(1.5%) control but different in the description; just to give you a few.

**Response**

Thank you so much for this observation. The interpretation has been revised using the results from the table 1 see Lines (178-190)

**Comment #3**

Kindly make sure all the odds in the regression table are in the same decimal places to correspond with what is in the table description.

**Response**

Thank you so much for this observation. The interpretation has revised in both the table 2 and the table description to match.

**Comment #4**

Kindly state the specific neurological symptoms identified in the study rather than the generalisation, as this statement could be misleading.

**Response**

Thank you for your guidance. We have replaced neurological symptoms with convulsions in the discussion Line: 219.

**Reviewer # 4 comments**

**Comment #1**

The authors seek to determine the risk factors for malaria related mortality among children in Mbale regional referral Hospital. The strengths of this paper lie in its data analytical methods. Generally, it is a good paper but we need to find factors that do not cut across both in the cases and controls. These are the main factors that are causing mortality. For example, in severe anaemia, we have 80% dying and 50% surviving. What could have caused the 80% in the cases to die that was not in the survivors? This could bring out the gist of paper well. It could be a prevailing illness, NCD etc. Otherwise, the risk factors affecting the cases are the very factors affecting the controls in comparable measure in this paper.

**Response**

Thank you for this insightful comment. We acknowledge that many risk factors for severe malaria are present in both cases and controls. However, our analysis focused on identifying factors that were **independently associated with mortality**, using multivariable logistic regression. This approach accounts for differences in prevalence between cases and controls and isolates factors that **increase the risk of death** rather than merely being common features of severe malaria.

**Specific comments:**

**Comment #2**

Abstract:
Line 28 - 30: is 300 out of 32,400 representatives enough of the data?

**Response**

Thank you for this comment. The sample size of 300 participants (100 cases and 200 controls) was determined based on calculation to ensure 80% power to detect an odds ratio of 2.0 for key risk factors, at a 5% significance level. While a larger sample could increase precision, the study was constrained by the available eligible records. Nevertheless, the calculated sample was sufficient to detect meaningful associations for the primary outcomes of interest

**Comment #3**

Introduction:
Line 43 – 46, 56-60: A lot of plagiarism. Please reference peoples work. Consider the whole paper. There several sentences or paragraphs that you need to reference.

**Response**

Thank you for this guidance. We have carefully reviewed the manuscript and added appropriate citations where required

**Comment #4**

Problem statement:
Line 72 – 78: I want to believe that your problem statement is derived from that fact the mortality rate at MRRH is 2.7% way higher than the 0.6% on national average, What are the additional factors? Make mention of the additional factors that are actually causing mortality.

**Response**

Thank you for this, the additional factors have been added: This persistently high rate suggests that additional factors such as delayed care seeking, severe anaemia among others, may be influencing outcomes and limiting the effectiveness of existing interventions. Lines (76-78)

**Comment #5**

Methods:
The methods look fine but 300 representatives enough for the study? You could increase it to give you enough power for the study.

**Response**

Thank you for this comment. The sample size of 300 participants (100 cases and 200 controls) was determined based on calculation to ensure 80% power to detect an odds ratio of 2.0 for key risk factors, at a 5% significance level. While a larger sample could increase precision, the study was constrained by the available eligible records. Nevertheless, the calculated sample was sufficient to detect meaningful associations for the primary outcomes of interest

**Comment #6**

Line 116: In your sample size calculation, were you supposed to use the prevalence of severe anaemia or prevalence of severe malaria? Check that because this could affect your actual sample size.

**Response**

Thank you for the comment. The sample size for this case–control study was calculated based on the prevalence of the **exposure (risk factor) among controls,** rather than the prevalence of severe malaria itself. This is standard practice for case–control studies, as all study participants already had severe malaria, and the primary objective was to determine whether specific risk factors for example severe anaemia was associated with death. Specifically, we assumed an expected prevalence of severe anaemia of 26% among controls, 80% power to detect an odds ratio of 2.0, and a 5% significance level, based on previous studies

**Comment #7**

Line 145 – 146: “Making accurate data extraction and analysis impossible outside” This sentence doesn’t make sense.
**Response**

Thank you for pointing out this. The statement has been made clear: We excluded children who were eligible but lacked essential information on symptoms, treatment, or outcomes, as accurate data extraction and analysis was not possible for these records. Line (146-147)

**Comment #8**

Results:
How do malaria cases relate with risk factors determined given these catchment areas in the 16 districts?

**Response**

Thank you for your comment. Cases were drawn from the hospital records representing catchment area of 16 districts. While the study did not perform district-level stratified analyses, the enrolled children reflect the population presenting to Mbale Regional Referral Hospital. The identified risk factors, therefore represent predictors of mortality among children with severe malaria in this broader catchment area

**Comment #9**

Line 182 - 183: It’s the outcome death case categorizes the cases but before death they have exactly the same factors affecting them just as the controls (survivors), again what special factor kills the cases? If you consider the same number of participants for cases and controls, chances are high that there is no difference how the risk factors affect these 2 groups.

**Response**

Thank you for this insightful comment. While cases (deaths) and controls (survivors) may initially share similar exposures or clinical features, our analysis aimed to identify **factors independently associated with mortality**. Using multivariable logistic regression, we adjusted for potential confounding between variables to isolate the factors that **significantly increase the risk of death,** even among children who otherwise present similarly to controls. For example; convulsions and loss of consciousness, as well as severe anaemia, were independently associated with higher odds of death, indicating that these factors have a **direct impact on mortality beyond their presence in survivors.** Therefore, although both groups may share common exposures, these specific clinical features distinguish cases from controls in terms of fatal outcomes.

**Comment #10**

Line 199: You talk of multivariant analysis but I don’t see it the results? How does severe anaemia relate to say, convulsions, loss of consciousness and high parasitaemia for example?

**Response**

Thank you for the observation. All key clinical variables, including severe anaemia, convulsions, loss of consciousness, and hyper parasitaemia, were independently assessed in a multivariable logistic regression model to identify factors associated with malaria-related mortality. This approach allowed us to account for potential confounding between interrelated clinical features.

**Comment #11**

Discussion:
Good discussion

**Response**

Thank you for the positive feedback
**Comment #12**

Conclusion:
Line 280 – 281: State the results in the conclusion

**Response**

Thank you for your guidance, the conclusion has been revised.

Thank you for taking the time to review our manuscript and provide valuable feedback
