# Supplementary material for: Risk factors for malaria-related mortality among children under five at Mbale Regional Referral Hospital, Uganda, 2020–2024: A case-control study
Source: PLOS Glob Public Health. 2026 Apr 20;6(4):e0006308. doi: 10.1371/journal.pgph.0006308 (PMC13094984; doi:10.1371/journal.pgph.0006308)
Supplement: Attachment — Submitted filename: Response to reviewers commments-Kwizera __.docx [file pgph.0006308.s005.docx]

March 1, 2026

Dr. Helen Howard

Staff Editor

PLOS Global Public Health

**RE: Risk factors for malaria-related mortality among children under five at Mbale Regional Referral Hospital, Uganda, 2020–2024: a case-control study**

Dear Dr. Howard,

We sincerely thank the reviewers for their constructive comments, which have helped improve the clarity and scientific quality of the manuscript. A detailed point-by-point response to the reviewers’ comments and Editor are attached, with corresponding revisions highlighted in the tracked-changes and clean versions of the manuscript.

We acknowledge the efforts of the reviewers and the Editor. For any further clarifications and information, please don’t hesitate to contact us.

Sincerely,

Patrick Kwizera (Corresponding Author)

**RESPONSE TO REVIEWERS’ COMMENTS**

**Reviewer #1 comments**

**Comment # 1**

Authors have well addressed the comments I raised earlier on. It has been found that delayed care-seeking is a top risk factor; a recommendation for community-level education or strengthening the Village Health Team referral system would be highly relevant as a conclusion.

**Response**

Thank you for your positive feedback and constructive recommendation. We agree that delayed care-seeking is a critical and actionable risk factor.

In response, we have strengthened the conclusions to explicitly reflect the need for community-level education and reinforcement of the Village Health Team (VHT) referral system.

- In the **abstract conclusion** (Lines 39–40), we now state: *“Early recognition of danger signs and prompt care-seeking could reduce pediatric malaria mortality in high-burden settings like Mbale.”*
- In the **main conclusion** (Lines 286–291), we have incorporated a clearer dual strategy approach: *“…Reducing malaria-related mortality in this setting may require a dual strategy: strengthening early recognition and prompt management of severe malaria at hospital level—through effective triage, continuous staff training, and reliable access to blood transfusion and antimalarials—while simultaneously improving community awareness of danger signs and reinforcing referral systems, particularly through Village Health Teams, to reduce delays in care-seeking…”*

**Reviewer # 2 comments**

**Comment #1**

A part of the manuscript claims the activity was 'non-research,” but later mentions IRB approval, which seems contradictory, and these points need clarification. The authors should explicitly specify whether the study was ethically exempt, approved, or classified as public health surveillance, so they can address this inconsistency.
**Response**

We thank the reviewer for this observation, we have made the ethical considerations clear in the manuscript, this is indicated on lines 167-175

**Comment #2**
Lastly, some sentences in the manuscript lack citations to support the authority of certain concepts, so the authors should provide citations to support these statements.

**Response**

Thank you for your observation, the table has been thoroughly reviewed.

**Reviewer #3 comments**

**Comment #1**

The authors have made a very thorough revision, addressing the previous concerns very well. Few minor points need to be revised to prevent potential misunderstanding by the readers:
 Line 45 to 46, for clarify, kindly indicate where exactly the malaria cases and death increased, from 2019 to 2023.

**Response**

We thank the reviewer for this helpful clarification. We have revised the statement in the Background section (Lines 45–46) to explicitly indicate that the increase in malaria cases and deaths occurred **globally**, as reported in the World Malaria Report 2024. The sentence has been modified to improve clarity and prevent potential misunderstanding by readers.

**Comment #2**

Line 60 to line 63. Kindly check what you meant by what severe malaria is “Severe malaria is defined by WHO as clinical or laboratory evidence of vital organ dysfunction, with impaired consciousness/ coma, haemoglobin levels below g/dL, acute kidney injury, respiratory distress, circulatory collapse/shock, acidosis, jaundice, or disseminated intravascular coagulation”.
It is missing the key component of what malaria is, that is the presence of plasmodium in the blood, and not just the complication you have listed.

**Response**

Thank you for this important clarification. We agree that the earlier version did not explicitly state that malaria requires the presence of *Plasmodium* parasites in the bloodstream, and instead focused primarily on the complications of severe disease.

We have revised the Background section to clearly distinguish between malaria as a parasitic infection and severe malaria as its life-threatening manifestation. Specifically, Lines 66–71 now read:

*“Severe malaria occurs when confirmed P. falciparum infection is accompanied by life-threatening clinical or* laboratory evidence of vital organ dysfunction. According to WHO, this includes impaired consciousness or coma, severe anemia (hemoglobin <7 g/dL), acute kidney injury, respiratory distress, circulatory collapse *or shock, metabolic acidosis, jaundice, or disseminated intravascular coagulation.”*

**Comment #3**

From line 185 to 189, kindly bring the percentage sign to the proportions in the description to distinguish between the absolute numbers and the proportions, just like your preceding format.

**Response**

Thank you for this helpful observation. We have carefully revised the Results section (Lines 185–206) to ensure consistent presentation of proportions using the percentage (%) sign, clearly distinguishing absolute numbers from percentages in line with the preceding format.

In addition, we reviewed and corrected subject–verb agreement throughout the paragraph and improved consistency in reporting (n, %) across all variables. To enhance clarity and avoid unnecessary duplication of table values, we streamlined the narrative by presenting key proportions for context without over-repeating all numbers already shown in Table 1. We believe these revisions improve clarity, consistency, and overall readability of the Results section.
