# Supplementary material for: Risk factors for malaria-related mortality among children under five at Mbale Regional Referral Hospital, Uganda, 2020–2024: A case-control study
Source: PLOS Glob Public Health. 2026 Apr 20;6(4):e0006308. doi: 10.1371/journal.pgph.0006308 (PMC13094984; doi:10.1371/journal.pgph.0006308)
Supplement: Attachment — Submitted filename: Response to reviewers commments.docx [file pgph.0006308.s006.docx]

March 24, 2026

Dr. Helen Howard

Staff Editor

PLOS Global Public Health

**RE: Risk factors for malaria-related mortality among children under five at Mbale Regional Referral Hospital, Uganda, 2020–2024: a case-control study**

Dear Dr. Howard,

We sincerely thank the reviewers for their constructive comments, which have helped improve the clarity and scientific quality of the manuscript. A detailed point-by-point response to the reviewers’ comments and Editor are attached, with corresponding revisions highlighted in the tracked-changes and clean versions of the manuscript.

We acknowledge the efforts of the reviewers and the Editor. For any further clarifications and information, please don’t hesitate to contact us.

Sincerely,

Patrick Kwizera (Corresponding Author)

**RESPONSE TO REVIEWERS’ COMMENTS**

**Reviewer #2 comments**

Line 177—A part of the manuscript claims the activity was "non-research." The authors should clarify what that means.

**Response:**
Thank you for this important comment. We have clarified this statement in the Ethical Considerations section to improve understanding. Specifically, we now explain that the study was conducted as part of a public health response and was classified as non-research because its primary intent was disease control and programmatic improvement rather than the generation of generalizable knowledge.

We further clarified that this determination was made in accordance with guidelines from the Uganda Ministry of Health and the US CDC, and that the activity met the criteria for public health practice as outlined in applicable federal regulations and international ethical guidance. The revised text explicitly states that the activity was exempt from Institutional Review Board (IRB) review on this basis. This is indicated on lines (169-183)

**Reviewer #3 comments**

The manuscript removed absolute numbers (n) and reported only percentages, obscuring denominators. Revise all data to standard n (%) format consistently.

**Response:**
Thank you for highlighting this issue. We have carefully revised the entire manuscript to ensure that all sociodemographic and clinical variables are consistently presented in the standard n (%) format. This is indicated on lines (186-204)

We thank you for your time in reviewing our manuscript
